# Costs and benefits of replacing preventive antenatal iron and folic acid with multiple micronutrients in 25 low- and middle-income countries

**DOI:** 10.1136/bmjgh-2025-020597

**Published:** 2026-05-13

**Authors:** John Hoddinott, Clayton Ajello, Robert E Black, Jennifer Busch-Hallen, Martin Mwangi, Dylan Walters, Filomena Gomes

**Affiliations:** 1 Division of Nutritional Sciences, Charles H Dyson School of Applied Economics and Management, and Department of Global Development, Cornell University, Ithaca, NY, USA; 2 Kirk Humanitarian, Salt Lake City, UT, USA; 3 Department of International Health, Johns Hopkins Bloomberg School of Public Health, Baltimore, MD, USA; 4 Maternal and Newborn Health and Nutrition, Nutrition International, Ottawa, ON, Canada; 5 Healthy Mothers Healthy Babies Program, Micronutrient Forum, Washington, DC, USA; 6 Health Economics Unit, Nutrition International, Ottawa, ON, Canada; 7 NOVA Medical School, Universidade Nova de Lisboa, Lisbon, Portugal

**Keywords:** Global Health, Health economics, Health policy, Maternal health, Nutrition

## Abstract

**Introduction:**

Micronutrient deficiencies during pregnancy have serious consequences for both mother and child; thus the longstanding standard of care in low- and low-middle income countries (LMICs) has been daily prenatal iron–folic acid (IFA) supplementation. While prenatal multiple micronutrient supplements (MMSs) provide additional significant benefits in comparison to IFA supplements, the view that MMS is too expensive has hindered national MMS adoption. However, increased competition, volume procurement and the use of advanced purchase commitments have significantly reduced the cost of MMS.

**Methods:**

Using new cost data, we estimate the benefits of replacing IFA with MMS in both health (averted low birth weights (LBWs), stillbirths and female neonatal mortality) and monetary (costs of averted LBW and death; total economic value; benefit–cost ratios) terms in 25 LMICs with the greatest burden of LBW. A number of scenarios describing different coverage and procurement cost scenarios are explored.

**Results:**

Replacing preventive antenatal IFA with MMS would avert 3 514 594 LBW births, 186 369 stillbirths and 218 914 female neonatal deaths over 5 years in these countries. Providing MMS to all pregnant women receiving at least one antenatal care visit averts 7 272 320 LBW, 473 471 stillbirths and 541 591 female neonatal deaths. The total cost of replacing IFA with MMS ranges from US$201.8 million to US$1.326 billion, equivalent to between 0.5% and 3.0% of current spending on efforts to reduce undernutrition. Using the most conservative estimate, this would generate US$7.19 billion in economic returns and a benefit–cost ratio greater than 10. The cost of averting a stillbirth or neonatal death ranges from US$497 to US$1306.

**Conclusion:**

Replacing prenatal IFA with MMS cost-effectively generates large health benefits.

**WHAT IS ALREADY KNOWN ON THIS TOPIC**

**What this study adds**

- We demonstrate that the view that MMS is too expensive is no longer correct.
- Providing MMS to all pregnant women in 25 low- and middle-income countries (LMICs) with the highest burden of low birth weight (LBW) who currently make at least one antenatal care visit would cost between US$87.7 and US$111.1 million per year; this represents less than 3.0% of current spending on efforts to reduce undernutrition in low and middle income countries.
- Doing so would avert 7 272 320 LBW, 473 471 stillbirths and 541 591 female neonatal deaths while generating large economic benefits ranging from US$7.19 to US$107.67 billion.

**How this study might affect research, practice or policy**

- These findings provide a compelling case for LMICs to transition from preventive IFA to MMS.

## Introduction

 Adequate nutrition, alongside quality antenatal care (ANC) during pregnancy is critically important for the health of mothers and their babies. Unfortunately, micronutrient deficiencies among women of reproductive age and pregnant women are widespread, particularly in low- and low-middle-income countries (LMICs).^1^ Micronutrient deficiencies during pregnancy have serious consequences, including impaired fetal growth, birth defects, preterm birth, premature rupture of membranes, maternal and child cognitive impairment and long-term metabolic disturbances.^2 3^

The longstanding standard of care has been to recommend that women consume an iron–folic acid (IFA) supplement (typically consisting of 30–60 mg of elemental iron and 400 μg of folic acid) daily during pregnancy to prevent maternal anaemia, puerperal sepsis, low birth weight and preterm birth.^4^ There is now, however, a compelling body of evidence demonstrating that multiple micronutrient supplements (MMSs)—such as the United Nations International Multiple Micronutrient Antenatal Preparation (UNIMMAP) which contains 10 vitamins and five minerals (including 30 mg of iron and 400 μg of folic acid) at recommended daily amounts for pregnant women—provide additional significant benefits in comparison to IFA supplements, including reductions in low birth weight (LBW) births, preterm births, small for gestational age newborns, stillbirths and in female neonatal deaths.^5^,^7^ In the present analyses, we will refer to the widely used and extensively studied MMS formula ‘United Nations International Multiple Micronutrient Antenatal Preparation’. While challenges with supply chains and concerns regarding low IFA supplement compliance are often given as a reason for not replacing preventive IFA with MMS, the first and foremost concern is the belief that MMS is too expensive. For example, Verney *et al*^8^ estimate that the unit cost of 180 tablets is 71% higher for MMS than for IFA. Thus, while several studies show that the economic benefits of replacing IFA with MMS are substantial (see, eg, Kashi *et al*^9^, Scott *et al*^10^, Larsen *et al*^11^ and Shekar *et al*^12^), the higher cost of MMS has been seen as a barrier to replacing preventative IFA with MMS.^13^

Increased competition, volume procurement and use of advanced purchase commitments (APCs)—a commitment to purchase a certain volume and a pre-payment of a significant portion of the total contract cost—have significantly reduced the finished product cost of MMS, making MMS pricing comparable to IFA supplements. Using these updated costs, we estimate the benefits of replacing IFA with MMS in both health (averted LBW, stillbirths and female neonatal mortality) and monetary (cost of averted low birth weight, cost of averted death; total economic value; benefit–cost ratios (BCRs)) terms. Thus, this paper aims to determine the costs and benefits of transitioning from preventive IFA supplementation to MMS in 25 LMICs with the greatest burden of LBW under different costing and coverage scenarios.

## Methods

### Time frame

We assumed a time frame of 7 years. The first 2 years are transition years when activities such as implementation research, training of health workers and creation of training and social behaviour change and communication materials take place. This is followed by 5 years of implementation of MMS in place of preventive IFA supplements.

### Estimating the number of women to receive MMS

Given the impact of MMS on reducing LBW births, we estimated the number of women to receive MMS in the 25 LMICs with the highest burden of LBW births, using the data reported by Okwaraji *et al*^14^ for the year 2020.

We assumed that women would begin to take MMS near the end of the third month of pregnancy; this assumption is consistent with the fact that the median initiation of ANC typically occurs at some point at the start or middle of the second trimester of pregnancy. This assumption is also consistent with the timing used in three randomised controlled trials that assessed the effects of replacing IFA with MMS (West *et al*^15^, SUMMIT^16^, Zeng *et al*^17^). Thus, the number of pregnant women that would receive supplements per year for these 25 countries is the number of women with live births *plus* the number of women with stillbirths *plus* the number of women with miscarriages between weeks 12 and 40 of pregnancy.

Okwaraji *et al*^14^ provide estimates of the number of low birth weight births per year and the prevalence of LBW births. These data allow us to calculate the number of live births per year for these 25 LMICs (live births=the number of LBW births divided by the prevalence of LBW births).

Hug *et al*^18^ provide data on country-specific prevalences of stillbirths (the birth of a baby with no signs of life at or after 28 weeks of gestation). As the prevalence of stillbirths equals the number of stillbirths divided by the number of births, the number of stillbirths per year can be calculated as: prevalence of stillbirths multiplied by the number of births.

To the best of our knowledge, national-level data on the prevalence of miscarriages between 12 and 28 weeks of pregnancy does not exist. While the overall prevalence of miscarriage is said to be about 15%, the vast majority of these occur in the first 2 months of gestation.^19^ Das *et al*^20^ used data on miscarriages taken from India’s nationally representative National Family Health Survey Five. Excluding women currently pregnant, they have data on 176 482 women who were pregnant at 12 weeks. Of these women, 1825 reported a miscarriage after 12 weeks, a prevalence of 1.03%. A previous review of the epidemiology of miscarriages estimated that only 1–2% occur after 12 weeks of gestation.^19^ Conservatively, we assume that the prevalence of miscarriage between 12 and 28 weeks is 2%.

Thus, the number of pregnant women per annum who potentially could receive MMS equals = (number of live births+number of stillbirths) × 1.020. We assume that this number does not change over the time frame we considered.

We considered two scenarios for coverage. The first is based on the current coverage of IFA supplements (ie, receipt of any IFA). This scenario assumes that health services switch out what they provide, discontinuing the provision of preventive IFA supplements and replacing it with MMS. The second scenario is based on the percentage of pregnant women who undertake at least one ANC visit (ANC1+). In this scenario, we would assume that the first time a woman undertakes an ANC visit, she is provided with a bottle of 180 MMS tablets. This scenario assumes that health services discontinue the provision of preventive IFA supplements (switching them to MMS), while also increasing coverage by providing MMS to pregnant women who previously had not received IFA. Country-level data on current coverage of receipt of any IFA supplements and the percentage of pregnant women with at least one ANC visit were taken from the Lives Saved Tool (‘LiST’), accessed from https://www.livessavedtool.org/ in January 2025. Thus, the number of women who would receive MMS is the:

1. Number of pregnant women per annum who potentially could receive MMS multiplied by the percentage of women currently receiving any IFA.
2. Number of pregnant women per annum who potentially could receive MMS multiplied by the percentage of women with one or more ANC visits

### Estimating costs

We considered four cost categories:

1. Commodity costs, the cost of MMS and IFA. For our analysis, we assumed these are provided in a bottle containing 180 tablets.
2. Out-of-pocket household costs. These are costs to pregnant women (money and time) associated with obtaining MMS.
3. One-off transition costs associated with moving from preventive IFA to MMS.
4. Programme/health system strengthening costs.

We used current (2025) US dollar prices paid by large-scale purchasers of MMS. Specifically, Kirk Humanitarian reports an executed contract price with their supplier of US$2.15 per 180-count bottle of MMS tablets produced to UNIMMAP specifications on a volume of 10 million bottles.^21^ Further, they report, based on shipping rates then in effect, that an average additional US$0.08/bottle is allocated for shipping and handling to anywhere in the world.

The cost of MMS is reduced because Kirk Humanitarian enters into an APC with its supplier. By committing to purchase at scale, it has allowed manufacturers to take advantage of economies of scale in production and thus reduces unit costs. In the absence of such an arrangement, UNICEF^22^ reports that 180 MMS tablets can be purchased for US$2.50.

Providing MMS means that it would no longer be necessary to purchase IFA supplements as an anaemia preventive measure. Put differently, the relevant cost for the tablets is not the cost of the MMS tablets but rather the difference in the cost of providing MMS rather than IFA supplements. As of March 2025, UNICEF, a large-scale purchaser of IFA supplements, reports a purchase price of US$1.10 for a 100-tablet bottle of IFA consisting of 60 mg of iron and 400 μg of folic acid.^22^ They do not report an analogous price for 180 tablets; we assumed that the IFA purchase price was US$1.10×1.80 = US$1.98. These prices are based on relatively small production runs of IFA without an APC. As with MMS, we included an additional US$0.08 per bottle for shipping from the USA and handling. We obtained estimates of procurement costs of IFA, produced at scale (a minimum production of one million bottles of IFA per year) and with an APC in place. This price per unit is US$1.50 plus US$0.08 for shipping and handling (Kirk Humanitarian, personal communication). We assume that MMS imported into a country is exempt from import duties.

Estimating the time and money costs associated with accessing supplements during pregnancy is challenging as these vary across settings. For example, they will be affected by the distance pregnant mothers need to travel to health posts, whether they walk, take public or private transport, and the time spent waiting to see a health professional. However, under the two scenarios we consider, mothers are already incurring these costs, either to receive IFA (our first scenario) or to attend a health post for an ANC visit. Under either scenario, there is no additional cost to mothers associated with receiving MMS.

The Healthier Pregnancies and Brighter Futures for Mothers and Babies Consortium^23^ divides transition costs into two categories: (1) pilot projects and implementation research (to determine whether or how best to make this change); and (2) all other transition costs, such as training of health workers, adjustment to supply chains (including the management of existing IFA stocks) and updating social behavioural change and communication materials. They estimate implementation research to cost a median cost of US$2.00 per pregnant woman currently receiving any IFA. They assume that all other transition costs are US$4.00 per pregnant woman currently receiving any IFA. We use these figures when calculating transition costs, assuming that the one-time transition costs are US$6.00 per pregnant woman currently receiving IFA.

There is no consensus on the inclusion of costs for strengthening ANC services.^8^ The World Bank’s 2017 ‘Investment Framework for Nutrition’ assumes that there are no additional recurrent costs associated with replacing IFA with MMS.^24^ Engle-Stone *et al*,^25^ Kashi *et al*^9^ and Larsen *et al*^11^ also make this assumption. The underlying argument is that while efforts to strengthen ANC are desirable, these are not necessary when replacing preventive IFA with MMS. An alternative argument is that the introduction of MMS represents an opportunity for strengthening existing ANC services, for example by improving nutrition counselling, women centred care, screening for anaemia and efforts to increase usage of ANC services. Global Investment Roadmap for MMS^8^ assumes that these costs are US$4.00 for the population of pregnant women who will receive MMS but do not currently receive preventive IFA, noting that these may decrease over time. Accordingly, for women not currently receiving IFA we modelled these strengthening costs as equalling US$4.00 per pregnant woman in the first 2 years of implementation of MMS in place of preventive IFA, US$3.00 per pregnant woman in years 3 and 4, and US$2.00 per pregnant woman in year 5 of implementation. We assumed that costs incurred to strengthen existing ANC do not have any impact on LBW, stillbirths or female neonatal mortality, although they very plausibly could have.

We do not account for the additional costs of IFA (or iron supplements) for the treatment of maternal anaemia. Those costs exist independently of whether there is MMS or preventive IFA supplements within standard ANC. Because we assume that there is no change in iron deficiency during pregnancy when transitioning from IFA to MMS, while countries will likely need to procure IFA or iron supplements when a full MMS programme is scaled, those costs are not part of the transition—they are already (or should be) part of existing standards of care.

### Estimating health benefits

We considered three health benefits associated with replacing preventive IFA with MMS: reductions in the prevalence of LBW; stillbirths averted; and reductions in female neonatal mortality. We use the magnitudes found in the Smith *et al*^5^ meta-analysis of 17 randomised controlled trials done in 14 low-income and middle-income countries, which compared MMSs (containing) IFA versus IFA alone. This meta-analysis reports a 12% reduction in the prevalence of LBW; an 8% reduction in stillbirths; and a 15% reduction in neonatal mortality among females. We did not account for the potential health benefits of efforts to strengthen existing ANC.

### Estimating economic benefits

We used the estimates found in Lomborg^26^ that every LBW birth averted generates US$1900 in discounted present value economic benefits. These are driven primarily through increases in labour productivity (partially through the economic benefits of greater education) and secondarily through reduced costs associated with infant illness and death.^27^

There are several methods for valuing an averted death, also referred to as the value of a statistical life (VSL). One, adapted from Hoddinott,^28^ is to calculate the present value of lost income arising from the death of an individual. For this sample of countries, this calculation values a death averted as US$8380. A second approach is to use contingent valuation methods, see for example, the study by Patenaude *et al*^29^; this produces a VSL of US$30 313 (in purchasing power parity dollars). A third approach used to provide estimates of the monetary value of lives lost during the COVID-19 pandemic values lives in terms of total lost earnings and the value of lost leisure time. For our sample of countries, this method produces a VSL of US$100 000.^30^ We refer to these estimates as low, medium and high VSL, respectively. Additional details on how these VSLs are calculated are found inonline supplemental appendix 1.

### Modelling LBW and deaths averted, economic costs and benefits

The number of LBW births averted per year is calculated as the number of LBW births per year multiplied by the reduction in LBW birth prevalence (12%), multiplied by the coverage level in each country. The number of stillbirths averted per year is calculated as the number of stillbirths per year, multiplied by the reduction in stillbirth prevalence (8%), multiplied by the coverage level in each country. The number of female neonatal deaths averted per year is calculated as the number of female neonatal deaths per year, multiplied by the reduction in female stillbirth prevalence (15%), multiplied by the coverage level in each country.

We modelled two coverage scenarios, see table 1: (1) Replacing preventive IFA with MMS and existing levels of IFA coverage, and (2) providing MMS at a level of coverage equivalent to the percentage of women who have at least one ANC visit (ANC1+). For each scenario, we assume a time frame of 7 years. The initial 2 years are transition years when all transition costs are incurred. This is followed by 5 years of implementation.

**Table 1:** Costing scenarios

Scenario	Coverage	Incremental cost of supplements	Transition costs	Strengthening costs	Costs borne by pregnant mothers
1A	Current IFA coverage	MMS (Kirk Humanitarian) price—IFA (UNICEF supply) price US$2.23–2.06	US$6 per woman currently receiving IFA	US$0	US$0
1B	Current IFA coverage	MMS (UNICEF supply) price—IFA (UNICEF supply) price US$2.58–2.06	US$6 per woman currently receiving IFA	US$0	US$0
1C	Current IFA coverage	MMS (Kirk Humanitarian) price—IFA (produced at scale) price US$2.23–1.58	US$6 per woman currently receiving IFA	US$0	US$0
2A	Current ANC 1+ coverage	MMS (Kirk Humanitarian) price—IFA (UNICEF supply) price US$2.23–2.06	US$6 per woman currently receiving IFA	US$0	US$0
2B	Current ANC 1+ coverage	MMS (UNICEF supply) price—IFA (UNICEF supply) price US$2.58–2.06	US$6 per woman currently receiving IFA	US$0	US$0
2C	Current ANC 1+ coverage	MMS (Kirk Humanitarian) price—IFA (produced at scale) price US$2.23–1.58	US$6 per woman currently receiving IFA	US$0	US$0
2D	Current ANC 1+ coverage	MMS (Kirk Humanitarian) price—IFA (UNICEF supply) price US$2.23–2.06	US$6 per woman currently receiving IFA	Per woman not currently receiving IFA: Year 1 and 2 of implementation: US$4 Year 3 and 4 of implementation: US$3 Year 5 of implementation: US$2	US$0
2E	Current ANC 1+ coverage	MMS (UNICEF supply) price—IFA (UNICEF supply) price US$2.58–2.06	US$6 per woman currently receiving IFA	Per woman not currently receiving IFA: Year 1 and 2 of implementation: US$4 Year 3 and 4 of implementation: US$3 Year 5 of implementation: US$2	US$0
2F	Current ANC 1+ coverage	MMS (Kirk Humanitarian) price—IFA (produced at scale) price US$2.23–1.58	US$6 per woman currently receiving IFA	Per woman not currently receiving IFA: Year 1 and 2 of implementation: US$4 Year 3 and 4 of implementation: US$3 Year 5 of implementation: US$2	US$0

ANC, antenatal care; IFA, iron–folic acid; MMS, multiple micronutrient supplement.

In coverage scenario 1—using IFA coverage values—there are one-time transition costs of US$6.00 per woman currently receiving IFA. This is incurred in years 1 and 2. In years 3 through 7, the cost of providing MMS rather than IFA is the price differential between MMS and IFA multiplied by the number of women currently receiving IFA. We undertake three calculations based on different assumptions about the incremental costs of replacing IFA with MMS: (1) The incremental cost of replacing IFA (costed using the price quoted in the current UNICEF supply catalogue plus shipping, US$2.06) with MMS (costed using the price currently paid by Kirk Humanitarian, US$2.23). This is US$0.17 per woman (US$2.23–2.06); (2) The incremental cost of replacing IFA (costed using the price quoted in the current UNICEF supply catalogue plus shipping, US$2.06) with MMS (quoted in the current UNICEF supply catalogue plus shipping, US$2.58). This is US$0.52 per woman (US$2.58–2.06); and (3) The incremental cost of replacing IFA (costed using the price quoted for producing IFA at scale, US$1.58) with MMS (costed using the price currently paid by Kirk Humanitarian, US$2.23). This is US$0.65 per woman (US$2.23–1.58).

In coverage scenario 2—using the ANC1+ coverage values—there are transition costs of US$6.00 per woman currently receiving IFA, all incurred in years 1 and 2. In years 3 through 7, the cost of providing MMS rather than IFA is the cost of MMS multiplied by the number of women with at least one ANC visit. We then subtract the cost of IFA multiplied by the number of women receiving IFA (because, those women, would no longer be provided with IFA). We then redo these calculations, adding in the ANC strengthening costs per woman who would receive MMS but is not currently receiving IFA in the first 2 years of implementation (years 3 and 4 in our 7-year time frame). In the subsequent 2 years, these are assumed to decrease to US$3.00 per woman and in the last year, to US$2.00 per woman. As we have three price differentials and two assumptions regarding strengthening costs, this gives us six aggregate costs for ANC1+ scenarios (2A–2F).

Across coverage scenarios 1 and 2 (IFA and ANC1+ coverage levels) we have nine cost estimates based on different coverage levels, estimates of the cost differential between MMS and IFA, and the inclusion (or not) of ANC strengthening costs.

We calculated the cost of an LBW birth averted as the total cost of replacing IFA with MMS divided by the number of LBW births averted. We calculated the cost of an averted stillbirth or neonatal death as the total cost of replacing IFA with MMS divided by the number of deaths averted.

The economic benefit of an LBW birth averted is the number of LBW births averted multiplied by the discounted present value of averting an LBW birth. The economic value of averting a death (stillbirth and neonatal female) is the total number of deaths averted multiplied by the monetary VSL (low, medium, high).

### Modelling and data

All data used in this study and the statistical programme used for modelling are available from the first author on request.

### Role of the funding source

This work was partly funded by Kirk Humanitarian and the Bill and Melinda Gates Foundation. The funders had no role in the design, data collection, analysis, interpretation or decision to publish this manuscript.

### Patient and public involvement statement

Neither patients nor the public were involved in the design, or conduct, or reporting, or dissemination of this research.

## Results

The 25 LMICs with the highest number of low birth weight births are listed in online supplemental appendix table A2.1. These countries account for 87% of all LBW births in LMIC.

Annually, there are 79 675 160 pregnancies in these 25 countries. Current preventive IFA coverage ranges from 4.7% (Democratic Republic of Congo) to 86.3% (Nepal). Mean IFA coverage is 32.9%. The percentage of pregnant women who have attended at least one ANC visit ranges from 40.0% (Sudan) to 98.4% (Burkina Faso) with a mean of 84%. Under the scenarios where coverage of MMS would be that of current IFA coverage, MMS would be provided to 29 459 062 women per annum. Under the scenarios where coverage of MMS would be that of the percentage of women with at least one ANC visit, MMS would be provided to 66 580 020 women per annum. Online supplemental appendix table A.1 disaggregates these numbers by country.

Over the 5-year period where MMS is provided, 3 514 594 LBW births are averted when IFA is replaced with MMS, and 7 272 320 LBW births are averted when coverage values are set at ANC1+. The number of stillbirths averted is 186 369 (coverage at current IFA values) and 473 471 (coverage at ANC1+). Female neonatal mortality is reduced by 218 914 (current IFA coverage) and 541 591 (coverage at ANC1+).

**Figure 1:**
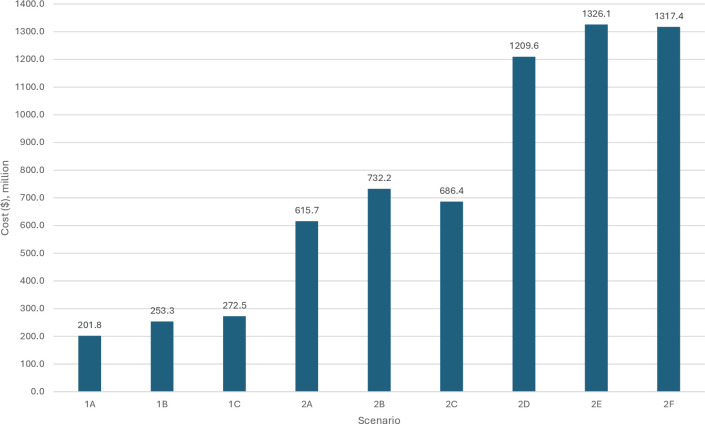
Total cost of replacing preventive IFA with MMS, in 25 LMICs over 7 years by scenario. IFA, iron–folic acid; LMICs, low- and middle-income countries; MMS, multiple micronutrient supplement.

Total costs over the 7-year period (2 transition years followed by 5 years of MMS provision) are reported in figure 1. These range from US$201.8 million (Scenario 1A: coverage at current IFA values; assume APC for acquisition of MMS; no strengthening costs) to US$1326.1 million (Scenario 2E: coverage at current ANC1+ values; assume no APC for acquisition of MMS; include strengthening costs). The contribution of each cost type (supplements, transition, strengthening) to total costs is reported in online supplemental appendix 3, online supplemental figure A2.1. Supplements, as a percentage of total cost, range from 12% (scenario 1A) to 76% (scenario 2B). Supplements make up a greater share of costs when coverage is assumed to be at the level of ANC1+ visits because some women receive MMS who would not have been receiving IFA; for these women, the cost of MMS is not offset by reductions in the cost of procuring IFA. Where we model the inclusion of the cost of strengthening ANC services, these strengthening activities account for 45–49% of all costs (online supplemental appendix 2, online supplemental table A2.2). Where MMS replaces IFA (1A, 1B, 1C), 65–88% of all costs are incurred during the transition phase. Supplement costs are higher (between US$87.7 and US$111.1 million per year) when coverage is at ANC1+ levels because a greater number of women are receiving supplements, including women who had not previously received IFA. Scenarios 2D, 2E and 2F have the highest yearly recurrent costs during implementation (between US$206.4 and US$229.8 million), driven by the costs associated with strengthening ANC.

**Figure 2:**
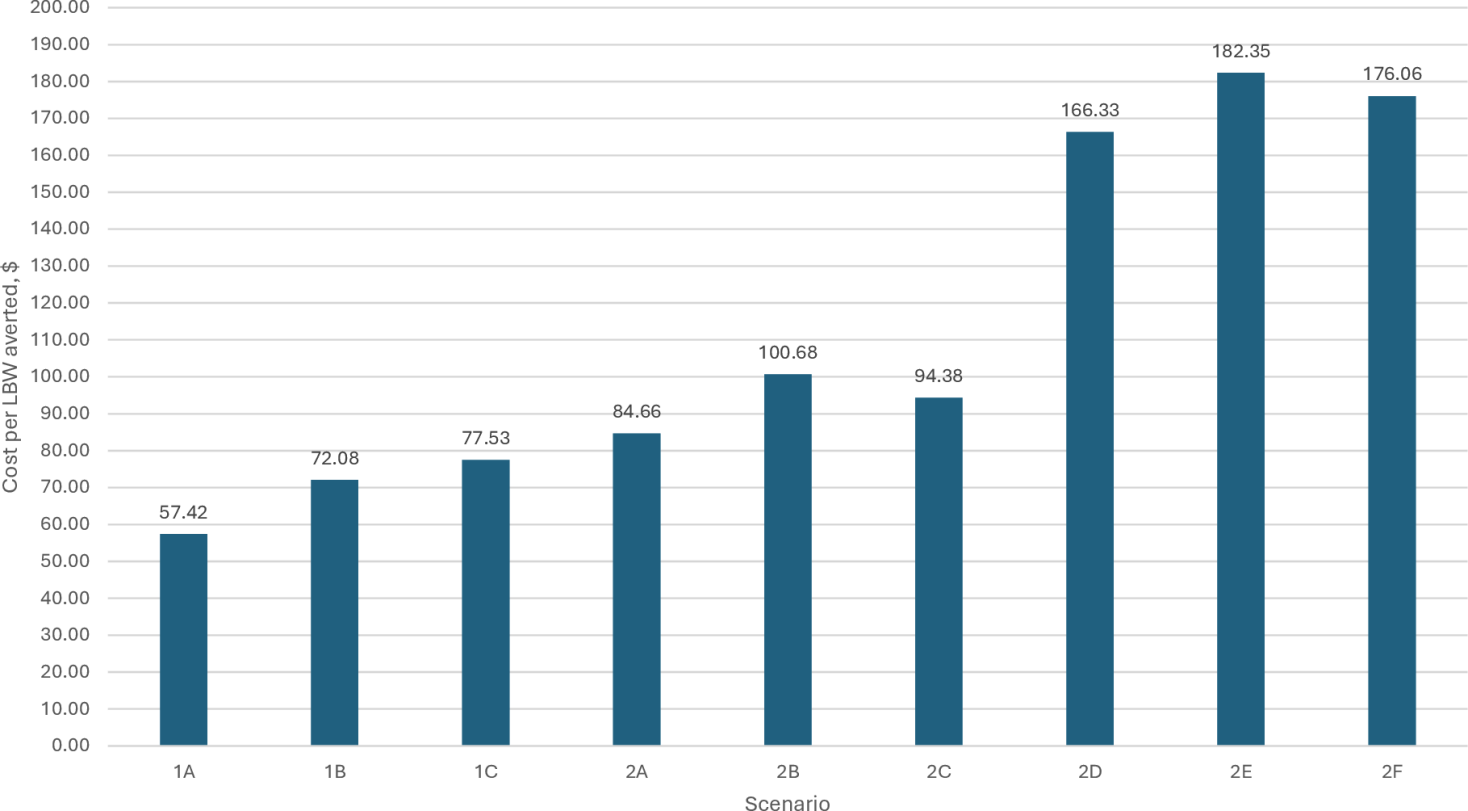
Replacing IFA with MMS, cost of LBW averted, over 7 years by scenario. IFA, iron–folic acid; LBW, low birth weight; MMS, multiple micronutrient supplement.

**Figure 3:**
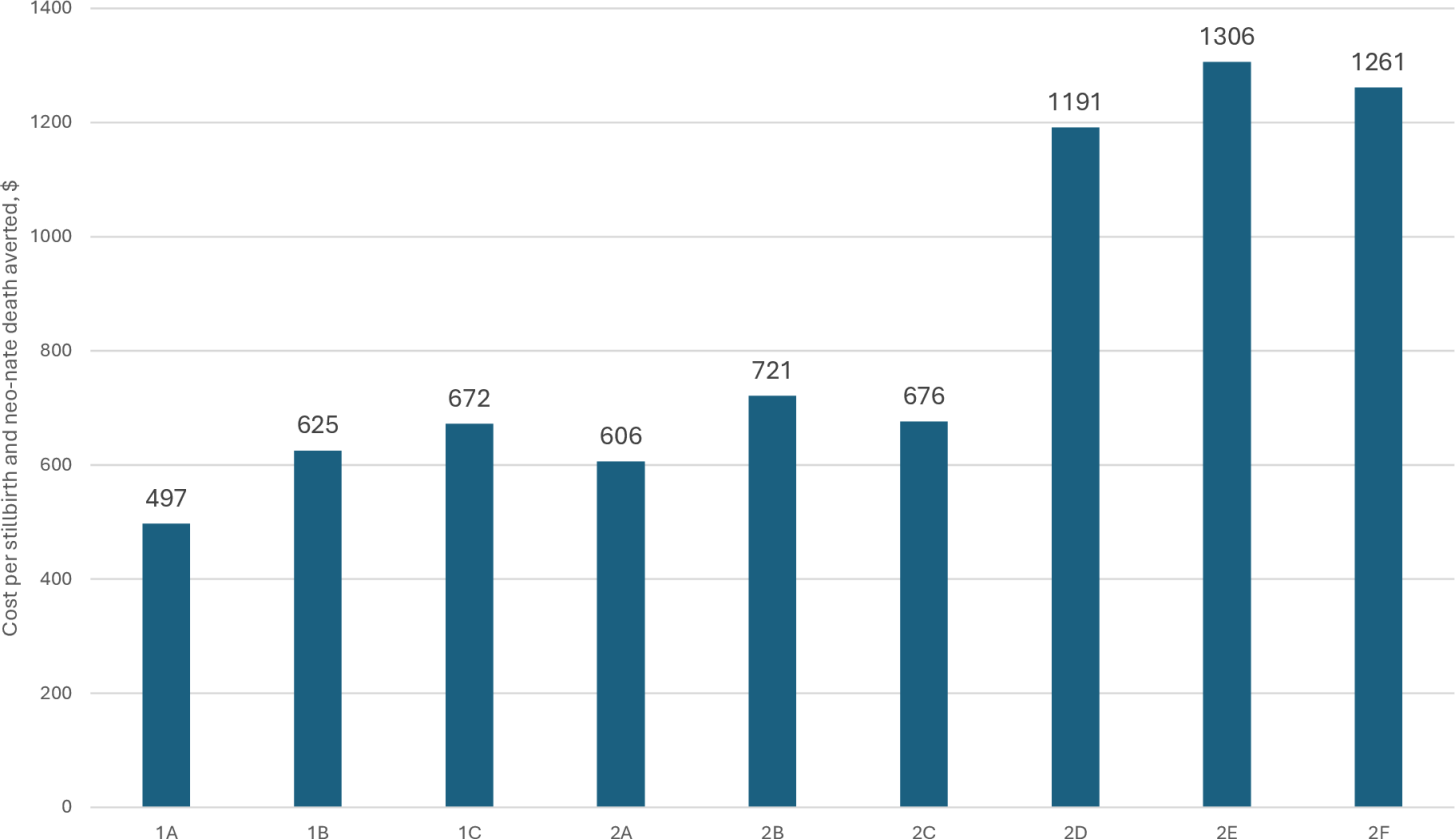
Replacing IFA with MMS, cost per stillbirth and neonatal death averted, over 7 years by scenario. IFA, iron–folic acid; MMS, multiple micronutrient supplement.

Figure 2 reports the cost per LBW birth averted. These range from US$57.42 (Scenario 1A) to US$182.35 (Scenario 2E). Figure 3 shows the cost of averting a stillbirth or neonatal death. These range from US$497 to US$1306. Comparing the ANC1+ coverage scenarios without and with ANC strengthening costs shows that adding these costs significantly increases the cost of averting an LBW birth or death, by 81–96%. The value of economic benefits reflects: (1) the number of LBW births and deaths averted, noting that these differ by coverage levels; and (2) the VSL used. Table 2 shows that the economic value of averted LBW birth is US$3.80 billion (IFA coverage) and US$6.16 billion (ANC1+) coverage. The economic value of deaths averted ranges from US$3.40 billion (IFA coverage, low VSL) to US$101.50 billion (ANC1+ coverage, high VSL). The lowest level of economic benefits is US$7.19 billion (IFA coverage) and the highest is US$107.67 billion (ANC1+ coverage, high VSL).

**Table 2:** Economic benefits, by scenario and value of statistical life

		Value ($ billion) of:										
									Benefit–cost ratios			
		LBW averted	Deaths averted			Total (LBW+deaths averted)			LBW averted only	LBW and deaths averted		
Coverage	Scenario		Low VSL	Medium VSL	High VSL	Low VSL	Medium VSL	High VSL		Low VSL	Medium VSL	High VSL
IFA	1A	3.799	3.396	12.285	44.327	7.195	16.084	48.127	18.8	35.7	79.7	238.5
	1B								15.0	28.4	63.5	190.0
	1C								13.9	26.4	59.0	176.6
ANC1+	2A	6.165	8.506	30.769	101.506	14.671	36.935.4	107.672	10.0	23.8	60.0	174.9
	2B								8.4	20.0	50.4	147.1
	2C								9.0	21.4	53.8	156.9
	2D								5.1	12.1	30.5	89.0
	2E								4.6	11.1	27.9	81.2
	2F								4.8	11.5	28.8	84.1

Notes: LBW is based on the discounted present value of economic benefits of averting an LBW. For VSLs: low VSL is calculated using present value of lost income; medium VSL is calculated using contingent valuation; and high VSL is calculated as trade-off between mortality risk and income. See online supplemental appendix 1 for additional details.

ANC, antenatal care; IFA, iron–folic acid; LBW, low birth weight; VSL, value of a statistical life.

BCRs reflect both the economic benefits of averting LBW births and deaths as well as the cost of each scenario. If we only consider the economic benefits of averting LBW births, BCRs range from 4.6 to 18.0. When the Low VSL value is used, BCRs range in value from 10.5 to 30.5. BCRs for medium VSLs range from 28.9 to 74.1 and for high VSLs, from 76.9 to 204.2.

## Discussion

We estimate that replacing preventive antenatal IFA with MMS would avert 3 514 594 LBW births, 186 369 stillbirths and 218 914 female neonatal deaths over 5 years in the 25 LMICs with the highest number of LBW births. At the time of writing (late 2025), once transition costs are taken care of (US$176.6 million over 2 years), the additional cost of replacing IFA with MMS is low, between US$5.0 and US$19.1 million per year. Providing MMS to all pregnant women receiving at least one ANC visit averts 7 272 320 LBW, 473 471 stillbirths and 541 591 female neonatal deaths. The marginal cost of doing so lies between US$87.7 and US$111.1 million per year.

The total additional cost of replacing IFA with MMS ranges from US$201.8 million to US$1.326 billion in the 7 years modelled. In the early 2020s, across all low and low-middle-income countries, US$6.3 billion per year is spent on addressing undernutrition in all its forms.^12^ The additional spending associated with the transition to MMS represents, over the 7 years considered here, between 0.5% and 3.0% of current spending on efforts to reduce undernutrition.

Valuations of economic benefits are sensitive to assumptions about the monetary value of a life saved. Using the most conservative estimate, replacing preventive IFA with MMS would generate US$7.19 billion in economic returns. Estimates of the BCRs are similarly sensitive to the valuation of life saved. That said, even using the highest estimates of costs (scenario 2E: coverage at ANC1+ levels and including strengthening costs) and the lowest valuations of lives saved (present value of lost income arising from the death of an individual) generates a BCR larger than 10. The cost of averting a stillbirth or neonatal death is low, ranging from US$497 to US$1306 per (stillbirth and neonatal) death averted. To put these in context, Savinkina *et al*^31^ report a lower bound estimate of the cost of averting a death through scaling up COVID-19 vaccinations in low- and low-middle-income countries to be US$7400. Isanaka *et al*^32^ report that the provision of ready-to-use therapeutic foods (RUTF) to treat moderate acute malnutrition in Mali at US$9821 per death averted. Rheingans *et al*^33^ estimate that a lower-bound cost per death averted for the rotavirus vaccine is US$3015. Using the LiST model, Eisele *et al*^34^ estimated that the scale-up of insecticide-treated bed nets and other malaria-prevention activities in sub-Saharan Africa between 2000 and 2010 cost US$2770 per death averted. Even under our most conservative costing scenario, the cost of averting a death through the provision of MMS is 82% less than the estimated cost of averting a death through scaling up vaccinations during the COVID-19 pandemic, 87% less than estimates of the cost of averting deaths through the provision of RUTF to moderately acutely malnourished children, 57% less than estimates of the cost of averting deaths through the provision of the rotavirus vaccine and 53% less than estimates of averting deaths through insecticide treated bed nets and related activities.

Our study has strengths. We use the recent price at which both MMS and IFA are being procured. We have credible estimates of the transition costs associated with replacing IFA with MMS. We consider two coverage scenarios: (1) replacing IFA with MMS; and (2) increasing supplementation coverage to match current levels of at least one ANC visit. We consider benefits in terms of both averted LBW births and averted deaths, either stillbirths or female neonatal deaths. Our estimates of the monetary value account for different approaches to calculating the VSL.

Our study also has limitations. Our calculations assume that the number of pregnant women is constant. If, because of population growth, more women enter into reproductive age over the time period we consider, costs will be higher. But if fewer women become pregnant because of reductions in fertility rates, costs will fall. We assume these offset each; but if they do not, they are unlikely to significantly affect our key findings. We assume that MMS imported into a country is exempt from import duties. Strengthening costs, while based on best available information, are poorly documented; improved understanding of these would be beneficial. It is not known whether ANC strengthening activities will improve the outcomes considered in this paper; omitting these underestimates the benefits estimated in this paper. We assume that the 180 bottle of MMS given early in pregnancy will result in sufficient use of the supplement to have the effects on LBW, stillbirth and female neonatal deaths assumed here. On this, we note that recent meta-analysis by Smith *et al*^35^ shows that the effect of MMS relative to IFA on birth weight is modified by supplement adherence with higher birth weights found where adherence was greater than 60%; they also find that adherence did not modify the relative effect of MMS compared with that of IFA on other infant outcomes, including stillbirths. We note that MMS is well accepted in terms of its organoleptic properties (size, taste/flavour, colour), see Kissell *et al*^36^ and that a recent trial assessing the adherence and acceptability of MMS in Cambodia showed very high adherence rates (mean 95%) to 180 tablets of MMS for women recruited at <14 weeks of gestation and where women received the prenatal counselling by trained healthcare workers, as per the current standard of care in Cambodia.^37^

When we model increasing supplementation coverage to match current levels of at least one ANC visit, we include women who would otherwise not have been receiving supplemental IFA. We assume that the benefits of doing so are the same as replacing supplemental IFA with MMS. If their effect sizes are larger, we will underestimate the benefits of expanding MMS coverage. If increasing supplementation coverage to match current levels of at least one ANC visit reduces anaemia in pregnant women, this would generate an additional benefit that we do not model.

We model transition costs as being incurred in the first 2 years of implementation, but some might extend beyond that. In our model, this changes when these costs occur, but not their total amount.

Our cost estimates are lower than those found in Shekar *et al*.^12^ One reason for this is that the primary data sources used by Shekar *et al*^12^ (including Young *et al*,^38^ Scott *et al*,^39^ and Kashi *et al*)^9^ use much older data on the cost of MMS supplements. For example, Scott *et al*^39^ assume 180 MMS tablets cost between US$5.52 and US$7.21. There are also differences in assumptions about how the provision of MMS instead of IFA affects the amount of time health workers spend counselling pregnant mothers. If health workers are fully employed and if no counselling currently takes place, the additional counselling time will require the hiring of additional health workers. The sources used in Shekar *et al*^12^ assume that these costs would be substantial whereas we assume that these already take place within existing standards of care, except in the scenarios where we include costs for strengthening ANC.

We make two additional comments: (1) There has been a large reduction in the cost of procuring MMS. Because the costs of procuring MMS are now similar to the cost of procuring IFA, the cost drivers underlying replacing IFA with MMS are largely transition costs and if implemented, ANC strengthening costs. This implies that efforts to further reduce the costs associated with this change should focus on developing cost-effective ways of reducing transition costs and (2) costs are a function of policy choices. Two are particularly salient. First, the transition from IFA to MMS represents an excellent opportunity to strengthen ANC care more generally, something that might be particularly important when scaling MMS beyond current coverage of IFA and to ensure that the challenge of adherence to the 180+ MMS recommendation is achieved. Doing so significantly adds to the cost of this transition but may also generate benefits not captured in our analysis. Second, historically antenatal supplements have been procured in small batches. This approach is costly because there are scale economies associated with the production of micronutrient supplements. Procuring at scale reduces the cost of producing supplements but procuring at scale requires manufacturers to be sure that there will be sufficient demand for the supplements that they produce. This is why the advanced purchase agreement or APC is crucial. Our estimates indicate that the APC for MMS reduces the cost of supplements by US$0.35 per pregnant woman, a 14% reduction. To put this into context, suppose a donor or government has a budget of US$50 million to purchase MMS. Without the advanced purchase agreement APC, this budget would provide MMS to 19.4 million women; with APC, this number is increased to 23.2 million.

Our findings provide a compelling *economic* case for LMICs to transition from preventive IFA to MMS.

## Supplementary material

10.1136/bmjgh-2025-020597 online supplemental file 1

10.1136/bmjgh-2025-020597 online supplemental file 2

## Data Availability

Data are available from the corresponding author upon reasonable request.
