# Supplementary material for: Costs and benefits of replacing preventive antenatal iron and folic acid with multiple micronutrients in 25 low- and middle-income countries
Source: BMJ Glob Health. 2026 May 13;11(5):e020597. doi: 10.1136/bmjgh-2025-020597 (PMC13182302; doi:10.1136/bmjgh-2025-020597)
Supplement: online supplemental file 1 [file bmjgh-11-5-s001.docx]

**Supplementary Appendix 1: Valuing averted deaths**

As noted in the text, three methods can be used to ascribe a monetary value to an averted death. These are described here.

***Present value of lost income***

This approach is an adaptation of that used in Hoddinott [1]. We begin with an estimate of current per capita incomes, specifically median per capita incomes reported in PPP dollars as reported in the World Bank’s World Development Indicators. An alternative approach, using mean per capita incomes, while feasible is problematic for countries with significant levels of inequality as it overstates the monetary benefit of saving a life. The median tells us how much the “typical” African or Asian earns (or consumes) in a year. We multiply each country’s median per capita income by each country’s share of LBWs and sum these weighted values. This sum equals $1,570 in 2022 PPP dollars.

Now consider a girl born in 2027 who is an averted stillbirth or neonatal death because her mother consumed MMS rather than IFA. Assuming that incomes grow by three percent per annum, her income when she enters the labor force at age 18 (year 2045) would be $2,835 PPP dollars. We then add up the income she would earn from ages 18 to 30 and discount this back to 2025 using a three percent discount rate, the same discount rate used in Shekar et al (2024). This yields a present value of income gained via averting a stillbirth or neonatal death equally $8,380 PPP dollars. This is the low VSL estimate.

Note that a higher discount rate would lower this amount; a lower discount rate would increase it. A higher assumed income growth rate would increase this amount; a lower assumed growth rate would lower it. Adding up income generated over a longer period would increase this amount.

***Contingent valuation***

Contingent Valuation (CV) approaches entail asking individuals how much they are willing to pay for a specified reduction in mortality risk, say for a specific disease affecting children (Keller et al, [2] 2021). Patenaude et al [3] apply this method in Tanzania, finding a willingness to pay of $9,340, or approximately 4.5 times the local per capita income. Trautmann et al [4] obtain similar results in Burkina Faso. Accordingly, we do the following. We obtain estimates of GDP per capita in 2022 Purchasing Power Parity US dollars for each country in our sample. We multiply these by each country’s share of LBWs and sum these weighted values. This sum equals $6,736 (PPP). We multiply this by 4.5, based on the results found in Patenaude et al [3] and Trautmann et al. [4]), yielding a VSL of $30,313. This is the medium VSL estimate.

Note that a smaller multiple of GDP would reduce this value; a larger multiple would increase it. The advantage of using PPP (rather than nominal US dollars) is that our results are less influenced by fluctuations in exchange rates while also accounting for differences in the costs of some goods and services across countries. Using nominal dollars instead of PPP dollars would lower this estimate of VSL.

***VSL as reflecting trade-offs between mortality risk and income***

A third approach to VSL, used extensively in high-income countries estimates the monetary value of a statistical life as the additional monetary amount that a worker is willing to accept in return for working in an occupation with a higher mortality risk [5]. An extension to this approach, described in Sweis [6], augments this by accounting for the value of lost non-work (“leisure”) time. Using her most conservative estimates, Sweis presents lower bound VSLs for eight countries that appear in our analysis. For six of these countries (India, Pakistan, Nigeria, Bangladesh, Indonesia, and Egypt), her lower bound estimate (in nominal 2022 US dollars) is $100,000. For two other countries, her lower bound estimate is $200,000 (Philippines) and $300,000 (Iran). We use her lowest estimate, $100,000. This is the high VSL estimate.

We note that for countries where VSLs derived from the first approach are absent, Viscusi and Masterman [7] generate estimates by looking at the association between country per capita income and VSLs for countries where such estimates are available. Doing so, they estimate an average VSL of $107,000 (2015 US dollars) for low-income countries and $420,000 for low-middle-income countries. Keller et al’s (2021) systematic review of papers assessing health investments in developing countries report a median VSL of $580 663. Our estimate, $100,000, thus, can be seen as a lower bound estimate of VSLs calculated in this way.

**References**

[1] Hoddinott, J. 2016. The economics of reducing malnutrition in Sub-Saharan Africa. Global Panel on Agriculture and Food Systems for Nutrition Working Paper 21

[2] Keller, E., Newman, J.E., Ortmann, A., Jorm, L.R. and Chambers, G.M., 2021. How much is a human life worth? A systematic review. *Value in Health*, *24*(10), pp.1531-1541.

[3] Patenaude, B.N., Semali, I., Killewo, J. and Bärnighausen, T., 2019. The value of a statistical life-year in Sub-Saharan Africa: evidence from a large population-based survey in Tanzania. *Value in Health Regional Issues*, *19*, pp.151-156.

[4] Trautmann, S.T., Xu, Y., König-Kersting, C., Patenaude, B.N., Harling, G., Sié, A. and Bärnighausen, T., 2021. Value of statistical life year in extreme poverty: a randomized experiment of measurement methods in rural Burkina Faso. *Population Health Metrics*, *19*, pp.1-18.

[5] Viscusi, W.K. 2018. *Pricing lives*. Princeton University Press.

[6] Sweis, N., 2022. Revisiting the value of a statistical life: an international approach during COVID‑19. Risk Management 24:259-272.

[7] Viscusi, W.K. and Masterman, C.J., 2017. Income elasticities and global values of a statistical life. *Journal of Benefit-Cost Analysis*, *8*(2), pp.226-250.

**Supplementary Appendix 2: Additional tables**

**Table A2.1: Country List, estimated number and prevalence of Low Birthweight, 2020**

| **Rank by number, LBW** | **Country** | **Estimated number, LBW** | **Estimated prevalence** | **% of all LBW in these 25 countries** | **Number women** **estimated to receive, IFA** | **Number women estimated to receive, 1+ ANC visit** |
| --- | --- | --- | --- | --- | --- | --- |
| 1 | India | 6,348,116 | 27.4 | 43.9% | 12,562,914 | 20,387,815 |
| 2 | Pakistan | 1,156,408 | 18.2 | 8.0% | 1,966,435 | 5,832,419 |
| 3 | Nigeria | 979,708 | 12.5 | 6.8% | 2,482,500 | 5,664,984 |
| 4 | Bangladesh | 696,681 | 23.0 | 4.8% | 1,459,132 | 2,375,037 |
| 5 | Ethiopia | 615,310 | 16.0 | 4.3% | 425,568 | 2,942,842 |
| 6 | Philippines | 522,941 | 21.1 | 3.6% | 1,429,944 | 2,189,521 |
| 7 | Indonesia | 449,641 | 9.9 | 3.1% | 2,055,316 | 4,539,406 |
| 8 | Egypt, Arab Rep. | 419,475 | 16.9 | 2.9% | 920,320 | 2,327,570 |
| 9 | Congo, Dem. Rep. | 399,157 | 10.2 | 2.8% | 193,523 | 3,392,821 |
| 10 | Afghanistan | 290,036 | 20.7 | 2.0% | 100,023 | 1,123,792 |
| 11 | Uganda | 268,260 | 16.2 | 1.9% | 388,025 | 1,674,000 |
| 12 | Sudan | 219,775 | 14.4 | 1.5% | 111,068 | 634,672 |
| 13 | Tanzania | 219,524 | 9.7 | 1.5% | 968,584 | 2,108,785 |
| 14 | Mozambique | 204,738 | 17.8 | 1.4% | 311,153 | 1,087,233 |
| 15 | Angola | 203,866 | 15.5 | 1.4% | 438,385 | 1,106,204 |
| 16 | Niger | 191,910 | 17.3 | 1.3% | 244,713 | 971,927 |
| 17 | Côte d’Ivoire | 167,755 | 18.3 | 1.2% | 273,775 | 913,222 |
| 18 | Madagascar | 165,568 | 18.7 | 1.1% | 184,398 | 806,399 |
| 19 | Iran, Islamic Rep. | 149,028 | 12.0 | 1.0% | 255,297 | 1,243,295 |
| 20 | Kenya | 145,174 | 10.0 | 1.0% | 835,904 | 1,484,033 |
| 21 | Burkina Faso | 143,472 | 18.5 | 1.0% | 433,804 | 794,903 |
| 22 | Yemen, Rep. | 132,370 | 13.1 | 0.9% | 52,750 | 733,219 |
| 23 | Ghana | 130,074 | 14.4 | 0.9% | 566,070 | 919,629 |
| 24 | Mali | 128,747 | 14.4 | 0.9% | 259,735 | 736,533 |
| 25 | Nepal | 118,902 | 19.7 | 0.8% | 539,727 | 589,760 |

Notes: Authors’ calculations based on data reported by Okwaraji et al (2024) for the year 2020.

**Table A2.2: Cost by year, cost type, and scenario, (USD Million), over 7 years in 25 LMICs**

|  |  |  | Scenario | | | | | | | | |
| --- | --- | --- | --- | --- | --- | --- | --- | --- | --- | --- | --- |
| Year | Phase | Cost Type | 1A | 1B | 1C | 2A | 2B | 2C | 2D | 2E | 2F |
| Y1 | Transition | Transition | 88.3 | 88.3 | 88.3 | 88.3 | 88.3 | 88.3 | 88.3 | 88.3 | 88.3 |
| Y2 | Transition | Transition | 88.3 | 88.3 | 88.3 | 88.3 | 88.3 | 88.3 | 88.3 | 88.3 | 88.3 |
|  |  |  |  |  |  |  |  |  |  |  |  |
| Y3 | Implementation | Total | 5.0 | 15.3 | 19.1 | 87.7 | 111.1 | 101.9 | 236.1 | 259.5 | 250.3 |
|  |  | Supplement | 5.0 | 15.3 | 19.1 | 87.7 | 111.1 | 101.9 | 87.7 | 111.1 | 101.9 |
|  |  | Strengthening | 0 | 0 | 0 | 0 | 0 | 0 | 148.4 | 148.4 | 148.4 |
| Y4 | Implementation | Total | 5.0 | 15.3 | 19.1 | 87.7 | 111.1 | 101.9 | 236.1 | 259.5 | 250.3 |
|  |  | Supplement | 5.0 | 15.3 | 19.1 | 87.7 | 111.1 | 101.9 | 87.7 | 111.1 | 101.9 |
|  |  | Strengthening | 0 | 0 | 0 | 0 | 0 | 0 | 148.4 | 148.4 | 148.4 |
| Y5 | Implementation | Total | 5.0 | 15.3 | 19.1 | 87.7 | 111.1 | 101.9 | 199.0 | 224.1 | 213.2 |
|  |  | Supplement | 5.0 | 15.3 | 19.1 | 87.7 | 111.1 | 101.9 | 87.7 | 111.1 | 101.9 |
|  |  | Strengthening | 0 | 0 | 0 | 0 | 0 | 0 | 111.3 | 111.3 | 111.3 |
| Y6 | Implementation | Total | 5.0 | 15.3 | 19.1 | 87.7 | 111.1 | 101.9 | 199.0 | 224.1 | 213.2 |
|  |  | Supplement | 5.0 | 15.3 | 19.1 | 87.7 | 111.1 | 101.9 | 87.7 | 111.1 | 101.9 |
|  |  | Strengthening | 0 | 0 | 0 | 0 | 0 | 0 | 111.3 | 111.3 | 111.3 |
| Y7 | Implementation | Total | 5.0 | 15.3 | 19.1 | 87.7 | 111.1 | 101.9 | 161.9 | 185.3 | 176.1 |
|  |  | Supplement | 5.0 | 15.3 | 19.1 | 87.7 | 111.1 | 101.9 | 87.7 | 111.1 | 101.9 |
|  |  | Strengthening | 0 | 0 | 0 | 0 | 0 | 0 | 74.2 | 74.2 | 74.2 |
|  |  |  |  |  |  |  |  |  |  |  |  |
|  |  | TOTAL | 201.8 | 253.3 | 272.3 | 615.3 | 732.3 | 686.3 | 1208.9 | 1325.9 | 1279.9 |

**Supplementary Figure**

**Figure A2.1: Allocation of costs, over 7 years by scenario**
