# Supplementary figures and images for: Costs and benefits of replacing preventive antenatal iron and folic acid with multiple micronutrients in 25 low- and middle-income countries
Source: BMJ Glob Health. 2026 May 13;11(5):e020597. doi: 10.1136/bmjgh-2025-020597 (PMC13182302; doi:10.1136/bmjgh-2025-020597)

Figure A2.1: Allocation of costs, over 7 years by scenario

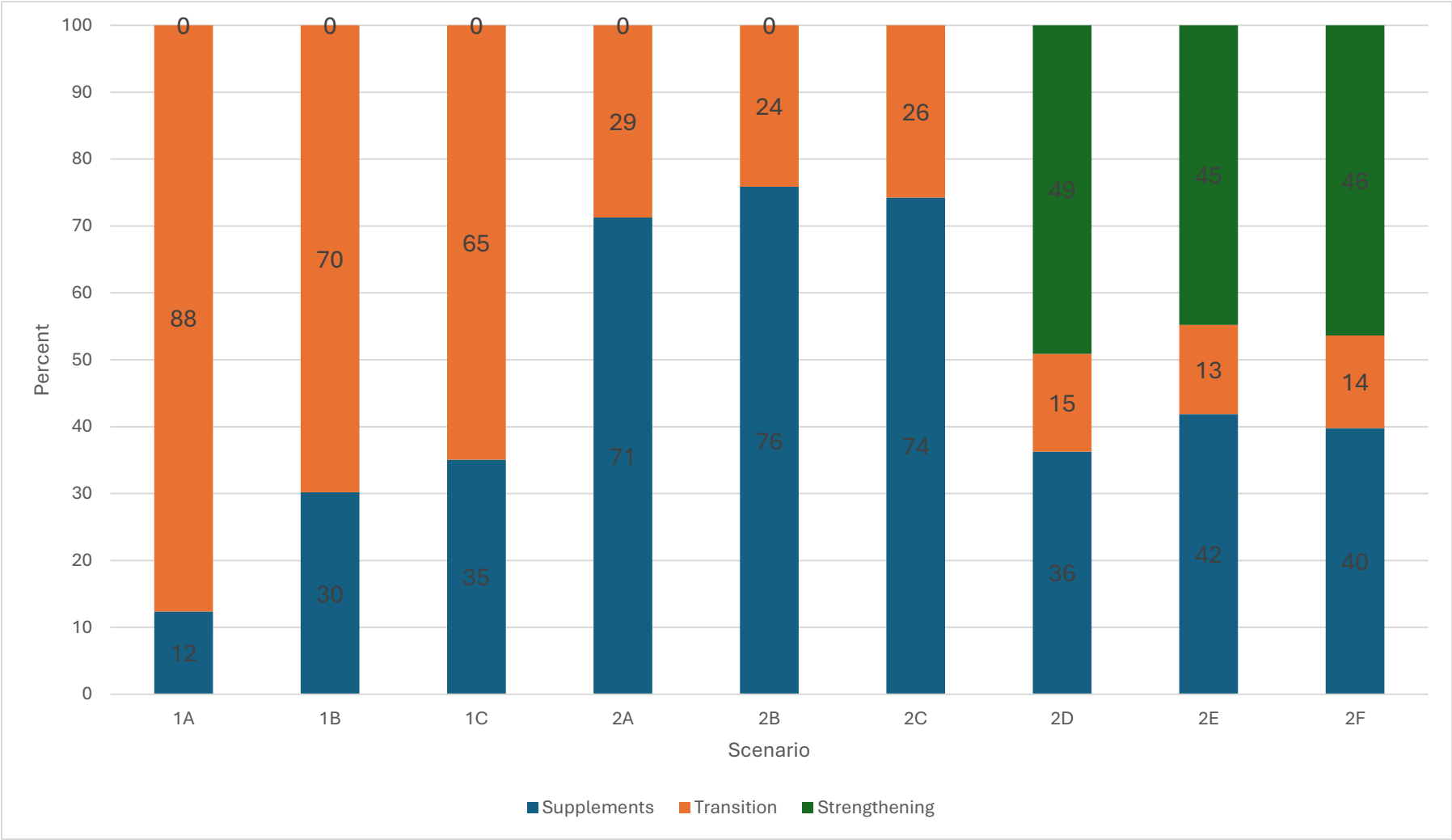

Supplement: online supplemental file 2 [file bmjgh-11-5-s002.pdf]
